# Small Cell Lung Cancer: State of the Art of the Molecular and Genetic Landscape and Novel Perspective

**DOI:** 10.3390/cancers13071723

**Published:** 2021-04-06

**Authors:** Valeria Denninghoff, Alessandro Russo, Diego de Miguel-Pérez, Umberto Malapelle, Amin Benyounes, Allison Gittens, Andres Felipe Cardona, Christian Rolfo

**Affiliations:** 1 National Council for Scientific and Technical Research (CONICET), University of Buenos Aires, Buenos Aires C1122AAH, Argentina; vdenninghoff@conicet.gov.ar; 2 Marlene and Stewart Greenebaum Comprehensive Cancer Center, University of Maryland School of Medicine, Baltimore, MD 21201, USA; alessandro-russo@alice.it (A.R.); ddemiguelperez@som.umaryland.edu (D.d.M.-P.); agittens@umm.edu (A.G.); 3 Medical Oncology Unit, A.O. Papardo, 98158 Messina, Italy; 4 Department of Public Health, University of Naples Federico II, 80138 Naples, Italy; umberto.malapelle@unina.it; 5 Thoracic Oncology, Inova Schar Cancer Center, Fairfax, VA 22031, USA; Amin.Benyounes@inova.org; 6 Clinical and Translational Oncology Group, Clínica del Country, Bogotá 110221, Colombia; andres.cardona@clinicadelcountry.com; 7 Foundation for Clinical and Applied Cancer Research (FICMAC), Bogotá 110111, Colombia; 8 Molecular Oncology and Biology Systems Research Group (Fox-G/ONCOLGroup), Universidad el Bosque, Bogotá 110121, Colombia

**Keywords:** small cell lung cancer, gene pathway, pathobiology, targeted therapy

## Abstract

**Simple Summary:**

Small cell lung cancer (SCLC) continues to carry a poor prognosis with a five-year survival rate of 3.5% and a 10-year survival rate of 1.8%. The pathogenesis remains unclear, and there are no known predictive or diagnostic biomarkers. The current SCLC classification as a single entity hinders effective targeted therapies against this heterogeneous neoplasm. Despite dedicated decades of research and clinical trials, there has been no change in the SCLC treatment paradigm. This review summarizes the body of literature available on SCLC’s genomic landscape to describe SCLC’s molecular/genetic aspects, regardless of therapeutic strategy.

**Abstract:**

Small cell lung cancer (SCLC) is a highly proliferative lung cancer that is not amenable to surgery in most cases due to the high metastatic potential. Precision medicine has not yet improved patients’ survival due to the lack of actionable mutations. Intra- and intertumoral heterogeneity allow the neoplasms to adapt to various microenvironments and treatments. Further studying this heterogeneous cancer might yield the discovery of actionable mutations. First-line SCLC treatment has added immunotherapy to its armamentarium. There has been renewed interest in SCLC, and numerous clinical trials are underway with novel therapeutic approaches. Understanding the molecular and genetic landscape of this heterogeneous and lethal disease will pave the way for novel drug development.

## 1. Small Cell Lung Cancer (SCLC) General Considerations

SCLC is a highly proliferative lung cancer that is not amenable to surgery in most cases due to the high metastatic potential. It is considered a high-grade neuroendocrine carcinoma with characterizing molecular alterations [1]. SCLC’s estimated five-year survival rate is 3.5%, and the 10-year survival rate is 1.8% [2]. Smoking history is present in 95% of the cases, and therefore carcinogenesis is linked to tobacco and its substrates, possibly through a DNA damage mechanism; however, the exact mechanism is unknown. The genes that affect oncogenes or tumor suppressor genes are usually acquired, not inherited. Tumor protein p53 (*TP53*) and retinoblastoma 1 (*RB1*) are the most common tumor suppressor genes (98% and 91%, respectively) [3]. These tumors are highly proliferative, as demonstrated by Ki67 immunohistochemistry [1,4,5,6,7]. SCLC is the deadliest lung cancer subtype and is uniformly fatal [8]. Lack of early detection and poor response to standard treatment are the main contributing factors to a poor outcome. SCLC usually responds to frontline therapy (60%–80% response rates); however, within 6–12 months, it becomes refractory to salvage treatments. Therefore, an understanding of resistance mechanisms is urgently needed. There has been renewed interest in SCLC, and numerous clinical trials are underway with novel therapeutic approaches. Understanding the molecular and genetic landscape of this heterogeneous and lethal disease will pave the way for novel drug development.

## 2. Molecular Pathways Involved in SCLC Development and Progression

**Figure 1:**
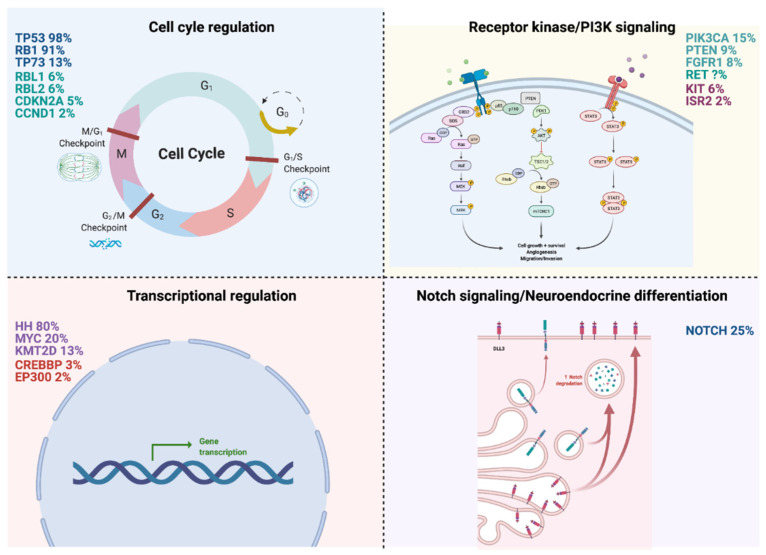
Signaling pathways recurrent affected in small cell lung cancer (SCLC) and frequently aberrant genes (created with BioRender.com, accessed on 28 March 2021).

Three pivotal comprehensive genomic analyses of SCLC shed light on SCLC development’s principle molecular pathways [9,10,11]. The limitation of these analyses is the small number of samples, most likely due to the lack of clinical specimens, as this disease is not usually treated with surgery. Therefore, experimental models and/or cell lines are fundamental for genomic analysis and sensitivity to treatments. Although *TP53* and *RB1* are the most common mutations found in SCLC, these alterations cannot yet be targeted pharmacologically. Peifer et al. sequenced 29 SCLC exomes, two tumor genomes, and 15 tumor transcriptomes. They observed a high mutation rate of 7.4 ± 1 protein-changing mutations per million base pairs; loss of TP53 and RB1; mutations and amplifications of *MYCL1, MYCN*, and *MYC*; mutations in the histone-modifying genes CREBBP, EP300, and *MLL*; mutations in *PTEN, SLIT2*, and *EPHA7*; focal amplification in *FGFR1* tyrosine kinase gene [9]. George et al. conducted whole-genome sequencing of 110 first frozen tumor samples from patients with limited and extensive-stage small cell lung cancer and their matched normal DNA [11]. They observed an elevated mutation rate of 8.62 non-synonymous mutations per million base pairs (Mb). C: G->A: T transversions were seen in 28% of all mutations and were linked to heavy smoking. The signaling pathways affected in SCLC and frequently aberrant genes in SCLC are shown in Figure 1.

SCLC neoplastic cells represent a broad molecular landscape. Thus, our current analysis techniques will detect the most frequent aberration within a given tumor sample. Intra- and intertumoral heterogeneity allow the neoplasms to adapt to various microenvironments and treatments. Further studying this heterogeneous cancer might yield the discovery of actionable mutations. Rubin et al. conducted a genetic study using RNA expression in mouse-derived SCLC cell lines and proposed a new classification. This classification identifies four main subdivisions based on the level of expression of *ASCL1* (achaete-scute homolog 1), classified as SCLC-A; *NEUROD1* (neurogenic differentiation factor one), classified as SCLC-N; *POU2F3* (pou class 2 homeobox 3), classified as SCLC-P; *YAP1* (yes-associated protein 1), classified as SCLC-Y. The expression of these four distinct genes has been established in both human (*n* = 81) and cell line tumor models (*n* = 54) [12]. The question is whether these molecular subtypes have different biologies and outcomes. Baine et al. studied protein expression by immunohistochemistry of these four molecular subtypes in a cohort of SCLC clinical specimens (*n* = 174). They also performed standard diagnostic stains, including neuroendocrine stains (SYP (synaptophysin), CgA (chromogranin A), CD56 (neural cell adhesion molecule 1), INSM1 (insulinoma-associated protein 1), TTF-1 (thyroid transcription factor 1), and DLL3 (delta-like ligand 3)) [13]. Based on the above results, the tumors were grouped into the following: ASCL1-dominant; NEUROD1-dominant; ASCL1/NEUROD1 double-negative with POU2F3 expression (POU2F3); ASCL1/NEUROD1 double-negative not otherwise specified (NOS) [13]. POU2F3 expression and the co-expression ASCL1/NEUROD1 were mutually exclusive. YAP1 was expressed in various subtypes and correlated with disease stage and survival. The authors suggested that YAP1 could be related to a transition phenotype between NSCLC and SCLC [13] and could induce multidrug resistance both in vivo and in vitro [14]. The SLCL-Y subtype seems to represent a well-differentiated tumor, with a marked inflamed microenvironment, rendering it perhaps more sensitive to immune checkpoint inhibitors [15]. DLL3 is absent in ASCL1/NEUROD1-negative tumors. This finding could be accounted for by the different techniques used across studies, protein vs. RNA analysis. These findings highlight the heterogeneity of SCLC. Identification of unique subtypes will allow the deployment of target treatments that will ultimately improve patient outcomes. Next, we review the genes and genomics/proteomic modifications related to the development, plasticity, and progression of SCLC, which could be identified as possible biomarkers for targeted therapy of this deadly disease.

### 2.1. Cell Cycle Regulation

#### 2.1.1. TP53/RB1 (98%/91%)

Biallelic loss of *TP53* and *RB1* has been found in 100% and 93% of cases, respectively, in extensive genomic studies. Other simultaneously occurring molecular alterations have been seen, such as mutations, translocations, loss of heterozygosity. However, biallelic loss of *TP53* and *RB1* remains an essential hallmark of SCLC carcinogenesis [11]. *TP53* mutations are missense mutations that are involved the DNA-binding domain. RB1 is affected by translocations and results in mutations in the exon–intron junctions, which leads to splicing events and subsequently damages proteins, as confirmed by transcriptome sequencing. *TP53* is located in 17p13.1 and has 12 exons. *TP53* encodes a tumor suppressor protein and can bind DNA and activate transcription. It plays a vital role in cell cycle arrest, apoptosis, and DNA repair. It is subject to alternative promoters, which results in multiple transcription variations. Many human cancers carry this mutated gene (Gene ID: 5925, updated on 7 February 2021) [16]. The mutations of *TP53* are numerous, but the clinically relevant substitutions in SCLC include Y220C, R248W, R249M, M237I, and R273L. *RB1* acts as a transcriptional corepressor, is located in 13q14.2, has 28 exons, negatively regulates the cell cycle, and stabilizes the chromatin structure. When activated, it binds to the transcription factor E2F1 (Gene ID: 5925, updated on 7 February 2021) [16,17]. Inactivation of RB1 can occur through different mechanisms: Point mutations, deletion, exon inversions, splice site mutations, and loss of mRNA expression [18]. Although neuroendocrine differentiation is a hallmark of SCLC, specific subtypes lack neuroendocrine differentiation. This might be relevant, as this subtype could be susceptible to CDK4/6 inhibitors and resistant to DLL3-targeted agents [18]. Neither *TP53* nor *RB1* are therapeutically targetable.

#### 2.1.2. TP73 (13%)

*TP73* (tumor protein p73) is located in 1p36.32 and has 16 exons. This gene encodes a member of the p53 family of transcription factors involved in cellular responses to stress and development. Many transcript variants resulting from alternative splicing and/or use of alternate promoters have been found for this gene. Still, the biological validity and the full-length nature of some variants have not been determined (Gene ID: 7161, updated on 22 March 2021) [16]. *TP73* is frequently altered in the SCLC genome (13%) [3,11,19]. The *TP73* alterations include gene rearrangements that result in NH-terminal truncation (p73Δex2 and p73Δex2/3) or COOH-terminal deletion (p73Δex10).

### 2.2. Receptor Kinase/PI3K Signaling

#### 2.2.1. PI3K3CA (15%)

The PI3K/AKT/mTOR pathway regulates cell cycle, proliferation, and survival. When activated, PIK3CA protein phosphorylates AKT, which leads to mTOR activation downstream and other factors such as CREB and PtdIns3P. In several solid tumors, the upregulation of the PI3K/AKT/mTOR pathway promotes carcinogenesis. Shibata et al. performed an extensive mutation screening of the *PIK3CA* gene and only found 3/13 (23%) mutations in SCLC cell lines and 2/15 (13%) mutations in samples of primary SCLC [20]. *PIK3CA* (phosphatidylinositol-4,5-bisphosphate 3-kinase catalytic subunit alpha) is located in 3q26.32 and has 23 exons. Phosphatidylinositol 3-kinase is composed of an 85 kDa regulatory subunit and a 110 kDa catalytic subunit. This gene has been found to be oncogenic and a pseudogene of this gene has been defined on chromosome 22 (Gene ID: 5291, updated on 22 March 2021) [16]. Missense mutations of *PIK3CA* mostly gain function and are located in the helical domain at G542, E545, and Q546 and the kinase domain H1047 in 80% of the cases. The most common mutation in *PIK3CA* is H1047R, which results in enzymatic over-activation. To evaluate the H1048 cell line (H1047R mutant) contribution of PI3K/AKT/mTOR signaling to SCLC cell proliferation, Umemura et al. used RNA interference to down-regulate the expression of *PIK3CA*, and a significant decrease in proliferation was observed [21]. PI3K inhibitors have been extensively used in clinical trials, but only a few have gained Food and Drug Administration (FDA) approval, mainly due to dose-limiting toxicities. Feng et al. recently published the effect of a Chinese medicinal formula, Baizhu Additive Powder (SLBZ-AP), on the pain control and survival of mice with metastatic lung cancer to the bone. It is postulated that SLBZ-AP partially exerts its effects through the PI3K/AKT/mTOR pathway [22].

#### 2.2.2. PTEN (9%)

*PTEN* (phosphatase and tensin homolog) is located in 10q23.31 and has 10 exons. It serves as a tumor suppressor gene and regulates the AKT/PKB pathway. Multiple translation initiation codons allow transcription by alternative splicing of numerous variants that encode different isoforms (Gene ID: 5728, updated on 7 February 2021) [16]. *PTEN* mutations are ubiquitous across a broad range of cancers and in 4%–9% of SCLC [3,23]. The function of *PTEN* in SCLC is not known. A revealing study was conducted by inactivating *PTEN* on an *RB1/TP53*-deleted mouse model that simulated human SCLC in a metastatic pattern and neuroendocrine features [24]. On the one hand, when a single *PTEN* allele was inactivated, SCLC progression occurred rapidly, indicating *PTEN*’s tumor-suppressing function in SCLC. On the other hand, homozygous *PTEN* inactivation synergized with *RB1,* and *TP53* loss promoted transformation from adenocarcinoma to neuroendocrine carcinoma [25].

#### 2.2.3. FGFR1 (8%)

The fibroblast growth factor receptor (FGFR) binds to the fibroblast growth factor (FGF) family. *FGFR1* (fibroblast growth factor receptor 1) is located in 8p11.23. It has 24 exons that encode an FGFR family member, where the amino acid sequence is highly conserved between members. Throughout evolution, they differ from one another in their ligand affinities and tissue distribution. *FGFR* has an extracellular ligand domain, a transmembrane domain, and an intracellular domain. The extracellular domain is composed of three immunoglobulin-like domains. The intracellular domain contains tyrosine kinase activity, setting in motion a cascade of downstream signals, ultimately influencing mitogenesis and differentiation [13]. Alternatively, spliced variants have been described; however, not all variants have been fully characterized (Gene ID: 2260, updated on 22 March 2021) [16]. It had been reported that a high copy number of the FGFR1 gene might be a possible therapeutic target [5,26]. Paracrine FGF signaling is described in SCLC and has a negative prognostic impact. Paracrine production of FGFs results in neo-angiogenesis in cancer cells through FGFR1 and FGFR2 [27]. However, aberrant FGFR signaling might only occur in the earlier stages of the disease. Biomarkers that assess FGFR inhibition response are missing and candidates are FGFR1 gene amplification, overexpression, or mRNA quantification [5]. To date, very few reports have been published on FGFR inhibitors in SCLC harboring FGFR signaling pathway aberrations [28].

#### 2.2.4. RET

*RET* (rearranged during transfection) is a proto-oncogene located in 10q11.21, has 20 exons, and encodes transmembrane tyrosine kinase protein receptor. When activated, it leads to the downstream activation of numerous pathways: RAS-MAPK, PI3K-AKT, and STAT3. The activation of this proto-oncogene can occur through both activating point mutations and cytogenetic rearrangement [29]. Chromosomal rearrangements involving *RET* have several fusion partner genes, for example: *KIF5B*, *CCDC6*, *CUX1* (Gene ID: 5979, updated on 7 February 2021) [16]. The prevalence of RET alterations in SCLC is unknown. The low prevalence of lack of surgical SCLC specimens renders the tasks of studying *RET* in SCLC difficult. Neither Peifer et al. nor Rudin et al. identified RET in SCLC as a statistically significantly mutated gene [9,10]. Dabir and colleagues performed Sanger sequencing on an SCLC metastasis and found an M918T mutation [30]. A skin biopsy from the same patient did not contain this mutation, establishing its somatic nature. The specimen also stained for RET by immunohistochemistry. Currently, basket trials for cancers with *RET* mutations are not enrolling SCLC patients.

### 2.3. Transcriptional Regulation

#### 2.3.1. Hedgehog Signaling Pathway (80%)

The Hedgehog (HH) pathway plays conserved roles in regulating a diverse spectrum of developmental processes: Cellular proliferation and differentiation [31,32]. The pathway is composed of three proteins: Sonic Hedgehog (SHH), Indian Hedgehog (IHH), and Desert Hedgehog (DHH). The pathway is associated with carcinogenesis; however, it has not been studied in depth in SCLC. HH appears to regulate stem cells that maintain and regenerate within adult tissues. Park et al. used a TP53/RB1 knockout mouse model and observed HH to be upregulated in SCLC independently of the pulmonary microenvironment. Activated Smoothened (sMO), a transmembrane protein part of HH, triggered clonality in human SCLC cell lines and appeared to initiate carcinogenesis in an SCLC mouse model. Deletion of *sMO* had the opposite effect [33]. HH signaling is important for the in vivo growth of SCLC, but the establishment of cell lines from SCLC tumors may lead to the loss of key HH pathway members’ expression [34]. This pathway is related to carcinogenesis, and therefore the discovery and synthesis of HH-specific signaling antagonists warrant further investigation [31]. On this basis, HH inhibition is a promising therapeutic target.

#### 2.3.2. MYC (20%)

*MYC* is a family of regulator genes and proto-oncogenes that encode for transcription factors, with three related human genes: *c-myc (MYC), l-myc (MYCL),* and *n-myc (MYCN). MYC* was the first gene to be discovered in this family. *MYC* (MYC proto-oncogene) is located in 8q24.21 and has three exons that encode a nuclear phosphoprotein. MYC is critical to cell cycle progression and apoptosis. *MYC* amplification is present in various human tumors, with 20% of SCLC (Gene ID: 4609, updated on 7 February 2021) [16]. *MYCL* (MYCL proto-oncogene) is located in 1p34.2 and has two exons (Gene ID: 4610, updated on 2 March 2021) [16]. *MYCN* (MYCN proto-oncogene) is located in 2p24.3 and has three exons that encode a protein with a basic helix–loop–helix (bHLH) domain. Multiple alternatively spliced transcript variants encoding different isoforms have been found for this gene (Gene ID: 4613, updated on 2 March 2021) [16]. SCLC is treated as a homogeneous disease without further molecular sub-classification. These tumors often acquire an *MYC* amplification (in one of the subtypes: *MYCL1* [9%], *MYC* [6%], or *MYCN* [4%]), dramatically accelerating tumorigenesis and metastatic potential [9,11]. MYC-amplified SCLC responds to frontline chemotherapy to only develop refractoriness and disease progression to subsequent lines of therapy. MYC’s effect on this subtype of SCLC’s natural history has not been confirmed in vivo yet [35]. Mollaoglu et al. studied an SCLC model with loss of *TP53/RB1* and elevated *MYC* expression [36]. This model was similar to the human one, as evidenced by elevated NEUROD1 and low neuroendocrine markers such as ASCL1. Animal models of SCLC with high levels of MYC are sensitive to aurora kinase inhibitors. Chalishazar et al. described that tumors with MYC overexpression are vulnerable to arginine deletion. Arginine deiminase (ADE-PEG 20) has been shown to have antineoplastic effects in mice with MYC-associated cancers [37]. Based on Rudin et al.’s molecular classification of SCLC, Ireland et al. used single-cell transcriptome analyses in both mouse and human models and observed that *MYC* plays a critical role in evolving the different SCLC molecular subtypes [12,38]. On the one hand, MYC triggers the transition of ASCL1+ to NEUROD1+ to YAP1+ subtype in neuroendocrine cells. On the other hand, MYC promotes POU2F3+ tumors from different cell types. Given SCLC’s intratumoral heterogeneity, it is assumed that this evolution happens in vivo as well. It is worth noting that MYC requires activation of the NOTCH pathway to induce carcinogenesis. Patel et al. recently reported that MYC and MYCL1 regulate the plasticity between these histological subtypes and molecular subtypes, then the role of the MYC family in SCLC tumorigenesis could be redefined to develop effective therapies [39].

#### 2.3.3. KMT2D (13%)

*KMT2D* (lysine methyltransferase 2D) is located in 12q13.12, has 56 exons, and is also known as MLL2 or MLL4. The protein methylates the Lys-4 position of histone H3. The encoded protein is part of a large protein complex called ASCOM, a transcriptional regulator of the beta-globin and estrogen receptor genes (Gene ID: 8085, updated on 16 March 2021) [16,23]. Most striking is the high frequency of truncating *KMT2D* mutations, which have been found in 17% of SCLC cell lines and 8% of SCLC tumors. Although truncating *KMT2D* mutations are occasionally homozygous, most are hemizygous, suggesting that decreased gene dosage may contribute to SCLC [40]. It is not clear whether KMT2D-mutant SCLC will benefit from therapeutic inhibition of the H3K4 demethylase lysine demethylase 1A (LSD1). Future work will need to determine which SCLC subsets are likely to benefit from current approaches to target chromatin dynamic states [41].

### 2.4. Notch Signaling/Neuroendocrine Differentiation

#### NOTCH (25%)

**Figure 2:**
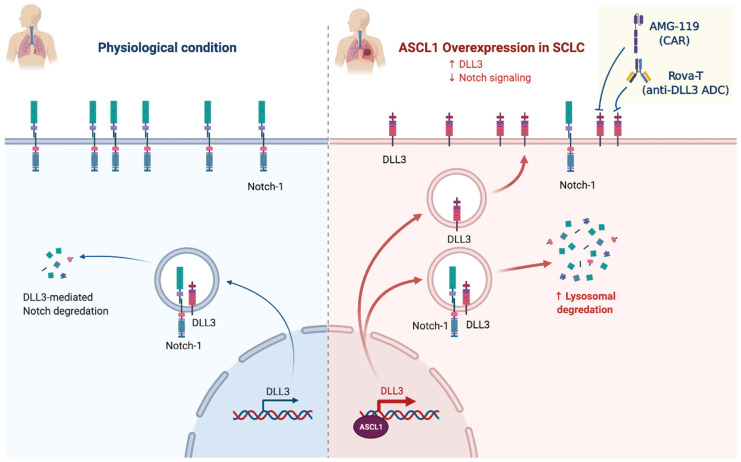
ASCL1 overexpression in SCLC and exploitation of DLL3 as therapeutic target (created with BioRender.com, accessed on 24 February 2021).

*NOTCH* receptor protein is a heterodimer transmembrane receptor that is proteolytically cleaved from a precursor protein (NOTCH1, NOTCH2, NOTCH3, or NOTCH4), and their fragment migrates to the nucleus. The ligand can be from within the same cell (cis-interaction) or from a different cell (trans-interaction) [42]. This fragmented protein in the nucleus is converted into a transcription regulatory protein inducing critical genes’ expression [43]. *NOTCH* mutation in SCLC is more commonly seen in the primary tumor rather than in the metastatic site. *NOTCH1* (NOTCH receptor 1) is located in 9q34.3 and has 34 exons that encode a member of this type I transmembrane protein family. This receptor is critical for developing various cells and tissues (Gene ID: 4851, updated on 7 February 2021) [16]. In SCLC, NOTCH1 signaling is suppressed and plays a tumor-suppressive role, is most widely mutated (25%), and the most mutations are missense mutations (82%). Mutations are associated with significantly improved survival [44]. Overexpression in *NOTCH1* inhibits SCLC growth and neuroendocrine features [45]. *NOTCH* negatively regulates the transcription factor ASCL1. On the one hand, ASCL1 promotes neuroendocrine transcription programs and is necessary for SCLC cells’ viability. On the other hand, when *ASCL1* is deleted in vivo, marked tumorigenesis inhibition is observed [46]. In general, the ASCL1 transcription factor is not targetable. However, LSD1, a lysine-specific histone demethylase 1, activates the NOCTH family upstream by suppressing *ASCL1* expression. SCLC highly expresses LSD1, which is attached to the *NOTCH1* gene [47]. Delta-like protein 3 (*DLL3*) is over-expressed in 80% of SCLC membrane cells and is specific to SCLC compared to normal lung cells. It is expressed both in the cytoplasm and in the membrane of SCLC cells [48]. Hence, DLL3 is a potential therapeutic target; clinical trials using a DLL3-targeted antibody–drug conjugate failed to benefit from toxicity concerns leading to discontinuation of the product. Other possible mechanisms to target DLL3 are illustrated in Figure 2 and include BiTE molecules^®^ (AMG757) and chimeric antigen receptor T cells (AMG119). AMG757 is a half-life extended bispecific T cell engager antibody construct that binds to DLL3 on cancer cells with one scFv domain and connects DLL3-positive cells to CD3-positive T cells, which causes tumor lysis and proliferation of autologous T cells (Phase 1 study NCT03319940) [49]. AMG119 is an autologous T cell that has been genetically engineered ex vivo to express a chimeric antigen T cell receptor directed toward DLL3 and results in tumor lysis and autologous proliferation T cells (Phase 1 study NCT03392064) [49].

Although *NOTCH3* expression in SCLC is lower than normal lung tissue [50], *NOTCH3* remains understudied, and further research is needed to determine its effect on SCLC biology.

### 2.5. Epigenetic and Proteomic Changes

How genetic and transcriptomic alterations affect the functional proteome in lung neoplasms is not fully understood. Epigenetics refers to ways to alter a phenotype’s expression that do not change the DNA sequence. It often occurs via methylation and histone modification [51]. *SLFN11* (Schlafen 11) epigenetic silencing, a putative DNA/RNA helicase, by the *EZH1*/*2* (Enhancer of the Zeste Homolog 1 or 2), has allowed us to gain an understanding of the role of epigenetics in SCLC. *SLFN11* seems to be a predictor of response to DNA-interfering agents such as topoisomerase I and II inhibitors, platinum, and PARP inhibitors [52]. For example, the clinical trial NCT03879798 was designed to evaluate whether EZH1/2 inhibitors could overcome chemotherapy resistance by reversing epigenetic silencing and restoring *SLFN11* expression [7]. Other clinical trials have used the bromodomain and extra-terminal motif protein (BET) inhibitor. These can modify the expression of several genes involved in carcinogenesis, such as *MYC*, *BCL2*, *CDK4*, and *CDK6*. The single-agent activity is limited but seems more promising in combination with other agents (NCT02391480) [7]. Stewart and colleagues studied 108 SCLC patients by mass spectrometry-based proteomics integrated with parallel analyses of DNA and mRNA to define molecular subtypes and identify drivers. With genomic, transcriptomic, and proteomic datasets, they identified three SCLC subtypes at the proteomic level. However, 87% of SCLC patients were associated with either immune infiltration (Inflamed) or oxidation-reduction (Re-dox) subtype [53].

### 2.6. Transcriptional Addictions

SCLC cells can manipulate and regulate gene expression to favor their growth and survival. Pharmacologically modulating gene expression could be a promising therapeutic approach. For example, on the one hand, THZ1 is a selective and potent covalent CDK7 inhibitor that suppresses SCLC growth. Christensen et al. demonstrated the efficacy of THZ1 treatment on the expression of proto-oncogenes such as *MYC* and neuroendocrine factors [54]. Meanwhile, on the other hand, lurbinectedin inhibits oncogenic genes’ active transcription, mainly in the GC-rich regulatory domains, and received the Food and Drug Administration’s (FDA) granted accelerated approval for extensive-stage SCLC after platinum-based therapy [55]. At the time of this manuscript’s submission, there is one ongoing clinical trial combining lurbinectedin with doxorubicin versus cyclophosphamide doxorubicin and vincristine for second-line SCLC after platinum based-therapy (NCT: 02566993).

## 3. Future Perspectives

SCLC has benefited little from the progress that the oncology field has seen in the last few decades. Diagnostically, a PET-radiotracer using 89Zr-SC16 is being developed. This radiotracer is directed toward DLL3; SCLC tracer uptake is correlated with DLL3 expression [7].

Bioinformatics strategy and extensive human sample collection will allow the study and discovery of potentially relevant molecular landscape and signaling pathways from a genomic perspective. Other potential areas of interest are epigenetic alterations in other genes (*CREBBP*, *KMT2D*/*MLL2*, and *MLL3*) and PIK3/mTOR pathway genes.

Although *PARP1* is overexpressed in SCLC, PARP inhibitors show little efficacy in SCLC with PIK3/mTOR pathway alterations. The same applies to *BCL2*. Although overexpressed as well in SCLC, BCL2 inhibitors show little benefit and significant hematological toxicity. Other DNA damage response proteins are also overexpressed in SCLC, such as *ATR* (ATR Serine/Threonine Kinase) [7].

Liquid biopsy is also a promising diagnostic tool that allows minimally invasive tumor genotyping and real-time monitoring [56]. Nong et al. performed deep-sequencing on 430 pretreatment SCLC biopsies and plasma samples from 22 SCLC patients at various treatment stages. They noted that average variant allele frequency is more predictive of survival than individual gene mutations, suggesting that clonal dynamics might be a vital determinant in SCLC biology [57]. Almodovar et al. developed a circulating free DNA (cfDNA) panel that detects 14 genes commonly mutated in SCLC [58]. They noted that most patients (85%) had genetic changes with mutant allele frequency between ≤0.1% and 84%, and *TP53* and *RB1* were most commonly mutated (70% and 52%, respectively). Interestingly, cfDNA allowed for relapse detection before this became evident radiographically. Liquid biopsy, therefore, has the potential of non-invasively tracking the disease status and response to treatment and provide valuable information before this becomes clinically evident. Carter et al. demonstrated that the circulating tumor cells were reliable in evaluating chemotherapy response and impacted progression-free survival [59].

## 4. Conclusions

Small cell lung cancer (SCLC) continues to carry a poor prognosis with a five-year survival rate of 3.5% and a 10-year survival rate of 1.8% [2]. The pathogenesis remains unclear, and there are no known predictive or diagnostic biomarkers. In this manuscript, we provided an overview of published studies on SCLC’s genomic landscape. Since there have been several comprehensive review articles published recently, this review summarizes the body of literature available on SCLC’s genomic landscape to describe SCLC’s molecular/genetic aspects, regardless of therapeutic strategy [3,4,10,60,61]. Further studies are needed to identify better genes and signaling pathways essential to SCLC cell survival and proliferation. Integration of preclinical and clinical data will be critical to understanding this lethal disease better. Bioinformatics is an integral part of this effort as it allows the analysis of SCLC “big data” in addition to next-generation sequencing, tumor genotyping, liquid biopsy, and transcriptomics. Once all of these techniques and efforts are assembled, it will be possible to develop novel therapeutic approaches to improve patient’s survival with SCLC.

## Data Availability

No new data were created or analyzed in this study. Data sharing is not applicable to this article.
